# Supplementary material for: Correlation holes and slow dynamics induced by fractional statistics in gapped quantum spin liquids
Source: Nat Commun. 2021 Mar 5;12:1459. doi: 10.1038/s41467-021-21495-8 (PMC7935948; doi:10.1038/s41467-021-21495-8)
Supplement: Supplementary file 1 — Supplementary Information [file 41467_2021_21495_MOESM1_ESM.pdf]

Supplementary Information for "Correlation holes and slow dynamics induced by fractional statistics in gapped quantum spin liquids"

Oliver Hart,[1] Yuan Wan,[2,3] and Claudio Castelnovo[1]

[1]T.C.M. Group, Cavendish Laboratory, JJ Thomson Avenue, Cambridge CB3 0HE, United Kingdom
[2]Institute of Physics, Chinese Academy of Sciences, Beijing 100190, China
[3]Songshan Lake Materials Laboratory, Dongguan, Guangdong 523808, China

(Dated: January 2021)

SUPPLEMENTARY NOTE 1: DERIVATION OF THE MICROSCOPIC MODEL

We focus our attention on a classical $\mathbb{Z}_2$ lattice gauge theory perturbed by a small, transverse magnetic field h. The model is composed of spin-1/2 degrees of freedom, $\boldsymbol{\sigma}_i$, which live on the bonds (labelled by the index i) of a square lattice with $N = L \times L$ sites (labelled by the index s) wrapped around a cylinder

$$H = -J \sum_s A_s - h \sum_i \sigma_i^z , \qquad A_s = \prod_{i \in s} \sigma_i^x . \tag{1}$$

Here $i \in s$ denotes the four spins that reside on the bonds surrounding the lattice site s. The coupling constant J ($\gg h$) is positive by convention. Treating the magnetic field h perturbatively, we arrive at the following ring-exchange Hamiltonian in the ground state sector:

$$H_{\text{eff}}^{(0)} = -J \sum_s A_s - \frac{5}{16} \frac{h^4}{J^3} \sum_p B_p \tag{2}$$

$$\equiv -\frac{\Delta_s}{2} \sum_s A_s - \frac{\Delta_v}{2} \sum_p B_p , \tag{3}$$

up to a constant energy shift that arises due to the virtual creation and annihilation of excitations. The plaquette operator B_p is defined as $B_p = \prod_{i \in p} \sigma_i^z$, where $i \in p$ denotes the four spins surrounding the plaquette p. The toric code Hamiltonian [1] is generated perturbatively and lifts the macroscopic degeneracy of the classical $\mathbb{Z}_2$ theory.

The ground state of the effective model, Supplementary Eq. (3), is characterised by eigenvalues +1 for all (commuting) operators A_s and B_p (and has a topological degeneracy that is immaterial for the purpose of the present work). Excitations correspond to states in which plaquette operators B_p and/or star operators A_s have negative eigenvalues. We will refer to the energetically costly star defects as spinons, and to the lower-energy plaquette defects as visons ($h^4/J^3 \ll J$, since $J \gg h$ by construction).

Let us then consider the two spinon sector, relevant for the intermediate temperatures of interest, $T \ll h, J$. The magnetic field h makes the spinons dynamical

$$H_{\text{eff}}^{(2)} = 4J - h \sum_{\langle ss' \rangle} \left(b_s^\dagger \sigma_{ss'}^z b_{s'} + \text{h.c.} \right) , \tag{4}$$

where $\langle ss' \rangle$ denotes neighbouring sites on the square lattice, and $\boldsymbol{\sigma}_{ss'}$ is the spin on the bond connecting sites s and s'. Since the magnetic field is applied parallel to the z axis, the vison configuration remains precisely static. The operators $b_s, b_s^\dagger$ are hardcore bosons representing the spinon excitations, which live on the sites of the lattice. Note that the spins $\sigma_{ss'}^x$ and the operators b_s are not independent: $A_s = e^{i\pi b_s^\dagger b_s}$. Crucially, each spinon hopping event is accompanied by a spin flip in the σ^x basis. In order to derive an effective tight-binding model, we make a gauge choice by fixing the string, $S(\gamma_i) = \prod_{\langle k\ell \rangle \in \gamma_i} \sigma_{k\ell}^z$, used to defined the state corresponding to a spinon residing on site i; the path γ_i ends on site i. Choosing a different string $S(\tilde{\gamma}_i)$ may lead to an additional phase: $e^{i\phi} = \langle S(\gamma_i) S(\tilde{\gamma}_i) \rangle$, $\phi = 0$ or π. Having made this choice, the hopping matrix element between adjacent sites i, j is given by $t_{ij} = -h \langle S(\gamma_i) S(\gamma_j) \sigma_{ij}^z \rangle$, where $\gamma_i \cup \gamma_j$ and the bond $\langle ij \rangle$ form a closed loop. Moving a spinon around a plaquette p, whose bonds are indexed by $\langle ij \rangle \in p$, the spinon acquires a phase

$$h^{-4} \prod_{\langle ij \rangle \in p} t_{ij} = \left\langle \prod_{\langle ij \rangle \in p} \sigma_{ij}^z \right\rangle = 1 - 2n_p . \tag{5}$$

The string operators do not appear in Supplementary Eq. (5) since $S(\gamma_i)^2 = \mathbb{1}$. Hence, for a given vison configuration, and when considering gauge invariant quantities, we can map Supplementary Eq. (4) onto a nearest neighbour tight-binding model

$$H_{\text{eff}}^{(2)}(\{\phi_{ss'}\}) = 4J - h \sum_{\langle ss' \rangle} \left(b_s^\dagger e^{i\phi_{ss'}} b_{s'} + \text{h.c.} \right) \tag{6}$$

$$\equiv \Delta_s \sum_s b_s^\dagger b_s - t_s \sum_{\langle ss' \rangle} \left(b_s^\dagger e^{iA_{ss'}} b_{s'} + \text{h.c.} \right) , \tag{7}$$

where the Peierls phases $\phi_{ss'} = -\phi_{s's}$ are, according to Supplementary Eq. (5), determined by the positions of the visons—each vison contributing a π-flux threading the plaquette on which it resides. With a cylindrical geometry, it is possible to choose a gauge in which the hopping amplitudes are real and uniform in one direction and acquire an appropriate minus sign in the orthogonal direction, according to the specific vison realisation [2]. Imposing periodic boundary conditions, the total flux threading all plaquettes must be an integer multiple of 2π, i.e., $\sum_p (\nabla \times A)_p = 0 \,(\text{mod}\ 2\pi)$.

SUPPLEMENTARY NOTE 2: COMPETING STRIP CONFIGURATION

As presented in the main text, the free energy of a spinon confined to a disc of radius ξ surrounded by disordered visons is given by

$$F_d(\xi) = \frac{j_0^2 t_s}{(\xi+\xi_0)^2} + \pi T \xi^2 \ln\left(1+e^{-\beta\Delta_v}\right) . \tag{8}$$

Neglecting the effects of nonzero ξ_0, and for temperatures satisfying $T \gg \Delta_v$, one arrives at the following expression for the radius ξ_* which minimises the disc free energy:

$$\xi_*^d = \left(\frac{j_0^2 t_s}{\pi T \ln 2}\right)^{1/4} . \tag{9}$$

For $\xi \gg \xi_0$, the disc model then predicts that the system will become completely free of visons at a temperature

$$T_*^d \simeq \frac{16 j_0^2}{\pi \ln 2}\frac{t_s}{L^4} . \tag{10}$$

In a square system of finite size with periodic boundary conditions, we observe an instability in our MC simulations whereby the shape of the depleted patch changes upon approaching T_*^d from a disc to a strip wrapping around the system. This is a finite size effect where the spinon wave function overlaps with itself across the periodic boundary conditions. It can be readily understood in light of the competition between the disc free energy and the free energy of a strip of width 2ξ in a system of size $L \times L$, with the corresponding vison strip free energy:

$$F_s(\xi) = \frac{\pi^2 t_s}{4(\xi+\tilde{\xi}_0)^2} + 2T\xi L \ln\left(1+e^{-\beta\Delta_v}\right) . \tag{11}$$

Notice that the strip width that minimises this free energy is, for $\xi \gg \tilde{\xi}_0$ and $T \gg \Delta_v$,

$$\xi_*^s = \left(\frac{\pi^2 t_s}{4TL\ln 2}\right)^{1/3} , \tag{12}$$

and the temperature at which the system becomes entirely free of visons is given by

$$T_*^s \simeq \frac{2\pi^2}{\ln 2}\frac{t_s}{L^4} . \tag{13}$$

In order to determine whether or not the system makes a transition from the disc state at high temperature to the strip state at low temperature, we compare the two free energies F_d and F_s. Solving for $F_d(T) = F_s(T)$, we find that the two free energies coincide at a temperature

$$T_{ds} = \frac{1}{4}\left(\frac{4}{3}\right)^6 \frac{j_0^6}{\pi \ln 2}\frac{t_s}{L^4} , \tag{14}$$

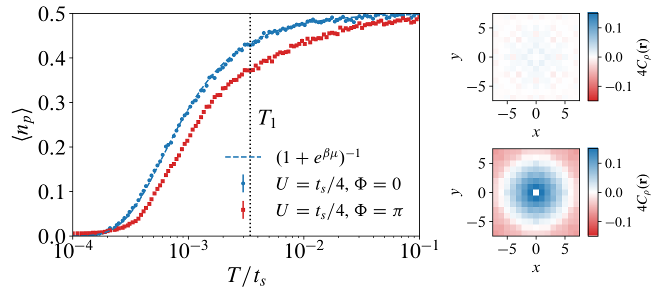

Supplementary Figure 1 | Effect of density-density interactions between spinons and visons. Left panel: vison density as a function of temperature in the absence (blue circles) and presence (red squares) of mutual statistics between the spinons and visons. The dashed blue line shows excellent agreement with the density of noninteracting visons in the presence of a uniform chemical potential μ, as discussed in the text. Right panel: corresponding vison correlations at the temperature T_1 indicated by the vertical dotted line on the left panel. In the absence of mutual statistics between the particles, the spinon only endows the visons with an effective chemical potential, but does not induce significant correlations between their positions. The data in both panels are for a system of size $L = 16$, averaged over 2^6 histories.

and that the disc free energy is lower than the strip one for $T > T_{ds}$. All of T_*^d, T_*^s, and T_{ds} scale with system size as $\propto L^{-4}$, and therefore the $O(1)$ prefactors determine whether or not an instability between the two vison configurations occurs. We find that $T_*^d, T_*^s < T_{ds}$, which implies that at the level of the saddle point approximation one expects a transition from the disc to the strip state at a temperature given by Supplementary Eq. (14), before the visons are eventually expelled from the system altogether below T_*^s.

Incidentally, the L^{-4} scaling with system size is indeed confirmed by the numerics in the inset of Fig. 2 in the main text, where the data for the vison density $\langle n_p \rangle$ are shown to collapse for various system sizes L when plotted as a function of TL^4/t_s.

SUPPLEMENTARY NOTE 3: SPINON–VISON INTERACTIONS

Here, we discuss the possibility that the Hamiltonian includes an explicit short-range interaction term between the spinons and visons. In particular, suppose that the spinons and visons are coupled by a density-density term of the form

$$H_{\text{int}} = H_{\text{eff}} + U\sum_s \left(\frac{1}{4}\sum_{p\in s} n_p\right) b_s^\dagger b_s . \tag{15}$$

That is, each spinon interacts with the four adjacent plaquettes p surrounding each site s (denoted by $p \in s$). The noninteracting Hamiltonain H_{eff} is given by Supplementary Eq. (7). We expect on rather general grounds that the interactions are repulsive $U > 0$ and weak $|U| < t_s$.

If the mutual statistics between the species is removed, i.e.,

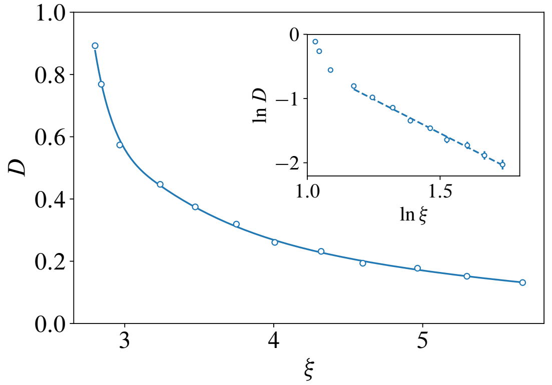

Supplementary Figure 2 | Evolution of the vison-depleted patch diffusion constant with its radius.. Dependence of the diffusion constant, D, on the vison-depleted patch radius ξ in equilibrium at temperature T, obtained from MC simulations, where time is measured in units of MC sweeps. The MC data in Fig. 2 of the main text are used to map between temperature and patch radius ξ. The solid line through the MC data is a guide to the eye. The inset shows that $D(\xi)$ is consistent with power law behaviour for sufficiently large ξ: $D \sim \xi^{-2} \sim \sqrt{T}$.

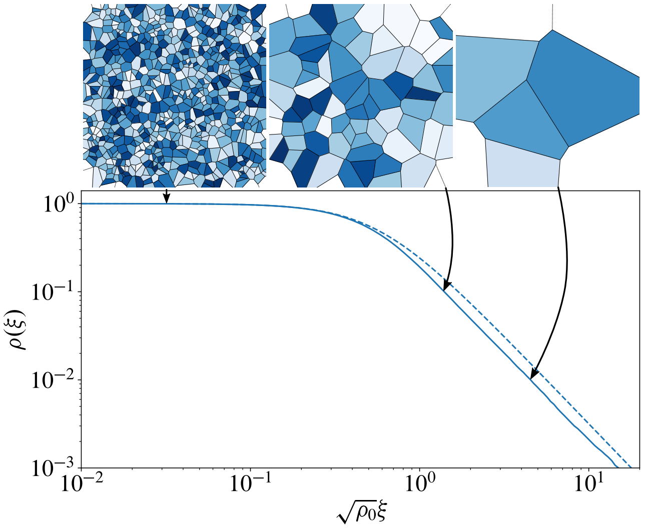

Supplementary Figure 3 | Evolution of vison-depleted patch density with patch radius. Numerical simulations of the effective patch model: as the radius ξ is increased, all neighbouring pairs of patches separated by a distance 2ξ or less are removed from the system. The centres of the patches remain fixed. The dashed line corresponds to the analytical result in Supplementary Eq. (18), valid in the dilute limit $\rho\xi^2 \ll 1$, and the solid line corresponds to the numerical results. The data correspond to 25 000 randomly distributed patches, averaged over 20 histories. The Voronoi diagrams show representative configurations of patches at densities ρ_0, $\rho_0/10$, and $\rho_0/10^2$ (left to right).

$A_{ss'} = 0$, then a typical configuration of visons gives rise to a diagonal (on-site) disordered potential term in the spinon tight binding Hamiltonian which also localises the spinons, as is generally expected in two spatial dimensions. However, since interactions are weak ($|U| < t_s$) the localisation length significantly exceeds the lattice spacing and we do not observe the formation of well-defined depleted patches. On the contrary, the vison density behaves smoothly, as if responding to a slowly-varying chemical potential with weak correlations between their positions. This behaviour can be seen in Supplementary Fig. 1, where the strength of the interactions is set to $U = t_s/4$. In these simulations, the localisation length exceeds the system size, and presence of a spinon translates into a uniform chemical potential $\mu \simeq U/L^2$ that controls the vison density as a function of temperature.

If the mutual statistics is reinstated, we observe the revival of the nontrivial correlations between the visons, implying the existence of a well-defined vison-depleted patch around the spinon. The linchpin of the formation of the vison-depleted patches and the implied nontrivial vison correlations is an effective disorder that gives rise to a localisation length (i.e., penetration depth) significantly smaller than the radius of the patch $\xi_{\mathrm{loc}} \ll \xi$, which eminently comes about as a result of mutual statistics and not of short-range spinon–vison interactions $|U| < t_s$.

SUPPLEMENTARY NOTE 4: DIFFUSIVE PATCH MOTION

In this section we study the dynamics of the vison-depleted patches as a function of temperature. Since there is no preferred direction for the motion of the patches, one generally expects them to perform an unbiased random walk across the system. Notice however that the motion of a patch over one lattice spacing requires the coordinated rearrangement of a number of visons which scales with the size of the patch. Hence, one expects the diffusion constant, D, (equivalently, the characteristic time scale) of the patch to decrease (grow) with decreasing temperature, as it involves the rearrangement of a larger number of visons.

Let us define the centre of the patch, $\mathbf{r}_p$, using the maximum of the spinon ground state wavefunction: $|\psi_0(\mathbf{r}_p)|^2 = \max_{\mathbf{r}} |\psi_0(\mathbf{r})|^2$. Asymptotically, one expects that $\langle \mathbf{r}_p^2(t)\rangle \simeq 2Dt$. This behaviour is indeed observed in the MC simulations, and the resulting values of D as a function of patch size ξ are shown in Supplementary Fig. 2.

Treating the patches as classical particles that satisfy the reaction-diffusion equation $A + A \rightleftharpoons \emptyset$, one may write down an equation for the evolution of the spinon density in the long-wavelength limit [3, 4]

$$\frac{\partial \rho_s}{\partial t} + \boldsymbol{\nabla}\cdot\mathbf{J} = -\mathcal{K}\rho_s^2 + \eta(\mathbf{r},t), \qquad (16)$$

where the current $\mathbf{J} = -D\boldsymbol{\nabla}\rho_s$, the annihilation constant $\mathcal{K} \propto D$, and $\eta(\mathbf{r},t)$ is a temperature-dependent source term that represents pairwise creation of the spinons at nonzero temperatures (with appropriate spectral properties). The rate of spinon annihilation in equilibrium is given by $\langle \mathcal{K}\rho_s^2\rangle$.

In order for the system to fall out of equilibrium as discussed in the main text, the cooling rate must be greater than the rate at which spinons can annihilate in order to remain in

equilibrium, i.e., $d\rho_s(T(t))/dt \gtrsim \langle \mathcal{K}\rho_s^2 \rangle$. At the mean field level (i.e., neglecting spatial fluctuations of the spinon density ρ_s), one may estimate the required cooling rate:

$$\left|\frac{dT}{dt}\right| \gtrsim \frac{DT^2}{\Delta_s} e^{-\Delta_s/T} . \tag{17}$$

Once again we see that, thanks to the exponentially small density of spinons in equilibrium at low temperature, this condition is likely to be easily (if not unavoidably) accessible experimentally in the study of quantum spin liquid materials.

SUPPLEMENTARY NOTE 5: EFFECTIVE PATCH GROWTH MODEL

Here we present an effective model of the growth of randomly distributed vison-depleted patches. The model represents a caricature of the dynamics of the system between temperatures $T_b \to T_c \to T_d$ in Fig. 4 in the main text, capturing both the plateau in the density of spinons between $T_b \to T_c$ and the kinematically-locked regime between $T_c \to T_d$.

Suppose that the initial density of patches is ρ_0 (equal to the equilibrium spinon density), and that their positions are random and uncorrelated. We assume that the centres of the patches remain stationary, whereas their radii are monodispersed at the typical equilibrium value, ξ, at temperature T. Then, the patches will grow as temperature is reduced until any two patches overlap, at which point the corresponding spinons annihilate. This process is described by the following mean field theory in the dilute limit, $\rho\xi^2 \ll 1$. When changing $\xi \to \xi + d\xi$ one can infer from geometric arguments that the reduction in the patch density is $d\rho = -8\pi\rho^2\xi d\xi$. One can then solve for the resulting density

$$\frac{\rho(\xi)}{\rho_0} = \frac{1}{1 + 4\pi\rho_0\xi^2} . \tag{18}$$

In the limit $\rho_0\xi^2 \ll 1$, the density remains essentially constant and unresponsive. This regime corresponds to the plateau between $T_b \to T_c$ in Fig. 4. The density begins to decay appreciably once a significant number of the patches start to touch, i.e., $\rho_0\xi^2 \sim 1$. This condition defines the temperature T_c, which represents the crossover between the plateau and the kinematically-locked regime. In the opposite limit, $\rho_0\xi^2 \gg 1$, the density of patches decays as $\rho \sim \xi^{-2}$, corresponding to the kinematically-locked regime between temperatures $T_c \to T_d$ in Fig. 4. Supplementary Eq. (18) implies however that $\pi\xi^2\rho = 1/4$ in this regime, which no longer satisfies the condition of diluteness that underpinned our simple modelling. Numerical simulations of the above effective model of patch growth (Supplementary Fig. 3) show that the relationship $\rho \propto \xi^{-2}$ predicted by Supplementary Eq. (18) does indeed hold in the regime $\rho_0\xi^2 \gg 1$, but with a modified prefactor, $\pi\xi^2\rho \simeq 1/6$.

[1] A. Kitaev, Annals of Physics **303**, 2 (2003).
[2] A. Furusaki, Phys. Rev. Lett. **82**, 604 (1999).
[3] V. V. Ginzburg, L. Radzihovsky, and N. A. Clark, Phys. Rev. E **55**, 395 (1997).
[4] A. Ovchinnikov and V. Atrazhev, Physica A: Statistical Mechanics and its Applications **276**, 1 (2000).
